# Open Data and transparency in artificial intelligence and machine learning: A new era of research

**DOI:** 10.12688/f1000research.133019.1

**Published:** 2023-04-12

**Authors:** Caellin M. Rodgers, Sally R. Ellingson, Parag Chatterjee

**Affiliations:** 1 F1000, London, UK; 2 UK College of Medicine, Markey Cancer Center, Lexington, Kentucky, USA; 3 Universidad Tecnologica Nacional Facultad Regional Buenos Aires, Buenos Aires, Autonomous City of Buenos Aires, Argentina; 4 Universidad de la Republica Uruguay, Montevideo, Montevideo Department, Uruguay

**Keywords:** artificial intelligence, machine learning, open data, open research, Artificial Intelligence and Machine Learning Gateway, sharing

## Abstract

Artificial Intelligence (AI) and machine learning are the current forefront of computer science and technology. AI and related sub-disciplines, including machine learning, are essential technologies which have enabled the widespread use of *smart *technology, such as smart phones, smart home appliances and even electric toothbrushes. It is AI that allows the devices used day-to-day across people’s personal lives, working lives and in industry to better anticipate and respond to our needs. However, the use of AI technology comes with a range of ethical questions – including issues around privacy, security, reliability, copyright/plagiarism and whether AI is capable of independent, conscious thought. We have seen several issues related to racial and sexual bias in AI in the recent times, putting the reliability of AI in question. Many of these issues have been brought to the forefront of cultural awareness in late 2022, early 2023, with the rise of AI art programs (and the copyright issues arising from the deep-learning methods employed to train this AI), and the popularity of ChatGPT alongside its ability to be used to mimic human output, particularly in regard to academic work. In critical areas like healthcare, the errors of AI can be fatal. With the incorporation of AI in almost every sector of our everyday life, we need to keep asking ourselves— can we trust AI, and how much?

This Editorial outlines the importance of openness and transparency in the development and applications of AI to allow all users to fully understand both the benefits and risks of this ubiquitous technology, and outlines how the Artificial Intelligence and Machine Learning  Gateway on F1000Research meets these needs.

## Open Science is becoming the new funding ‘norm’

Many large-scale funding bodies have, or are moving towards, new Open Science mandates in their funding requirements, including the European Commission ( European Commission, 2022) and the US government ( National Institutes of Health, 2023). To meet these requirements, all data utilised in research outputs should conform to the FAIR principles, ( Wilkinson *et al*., 2016), meaning that it should be **F**indable, **A**ccessible, **I**nteroperable and **R**eusable. In addition to Open data, to meet Open Science requirements, manuscripts should be published Open Access and underlying software or code utilised in producing the research output should be produced using an open-source option where possible.

For many researchers working in the field of Artificial Intelligence (AI), these concepts are not foreign – for example, the open-source operating system Linux ( Wilson *et al*., 2016) is a popular choice amongst computer scientists, and many share their codes freely on sites such as GitHub, BitBucket or similar. For others, especially those with more industry-focused roles who are often working to achieve patents, Open Science, and in particular data sharing, may be a newer concept. Luckily, there are plenty of resources freely available to help prospective authors with the new requirements of their funding bodies, including F1000’s data policies ( Grant, 2022).

The European Commission ( European Commission Publications Office, 2018) states that Open Data and data sharing are particularly important for AI and machine learning research due to the large volumes of data required to train machines up to a usable standard. This is particularly notable with respect to the recent rise of AI art, which is trained on art without the permission of the original artists ( Ghosh & Fossas, 2022), and may occur in other disciplines as well without either freely shared data to train the AI on and/or modern copyright legislation to protect others’ intellectual property rights.

## F1000’s publication model as a means of addressing Open Science requirements

Unlike many traditional publication models, the F1000Research model is designed specifically for the free sharing of research and its associated materials. It does this in four main ways.

Firstly, through use of Open Access – all articles published on F1000Research are freely available for all at point of publication. By removing the paywalls of traditional publishing, it means that researchers in under-funded countries or institutions also have access to valuable resources and reference work. Additionally, and of particular importance to AI research, news reporters and the general public also have access to this work, giving them the ability to read and understand discussions around AI directly from the source.

Secondly, F1000Research has strict Open Data mandates in line with the current updates to various funding mandates listed above. These mandates require that all data produced or used as part of a research work is available to peer reviewers, readers and researchers interested in reproducing or verifying the research. F1000Research operates under an ‘as open as possible, as closed as necessary’ policy, meaning that all material, including material that can’t be shared (for example, because it contains sensitive patient data) must be declared in the data availability statement along with any reason why it is unable to be shared. More available data is generally excellent news for researchers working in machine learning!

Thirdly, the publication model for F1000Research allows for a huge range of article types across the full research spectrum to be published. This means that valuable studies don’t have to wait until they’re ready to be turned into an original research article to start sharing data – methods articles, data notes and similar are all valuable ways that researchers can share their work prior to the final research article. The model also allows for versioning, which is great for researchers working in AI as it allows for updates to be made to articles as the field evolves, rather than requiring a full new article with only a minor improvement.

Finally, F1000Research employs Open Peer Review. This has several benefits to authors, readers and peer reviewers. For authors, it means that their work can be published online faster as peer review – typically the slowest part in the publication process – occurs after publication. For readers, it means that the full discussion about the article’s merits (and potential oversites or flaws) is freely available to read, allowing them the autonomy to decide for themselves about the article’s quality. For the reviewers themselves, it means they receive full credit for the work they put into this extremely important part of the research publication process, as their review is also published online and can be read and cited.

All these aspects are crucial for fostering an open, sharing research culture, essential to many new funder mandates and incredibly useful for AI researchers who often require a wealth of freely available data for quality machine learning.

## The future of the Artificial Intelligence and Machine Learning Gateway

Artificial intelligence and machine learning will continue to be at the forefront of the development of new computer-based technologies for the foreseeable future. However, with the issues arising from unconsidered applications, like students using ChatGPT to write homework essays ( Cotton *et al*., 2023) and the potential for a rise in AI-generated research articles with very little oversight ( Dergaa *et al*., 2023), it is important to share as much research and data openly as possible. This allows users of the technology to fully understand the work and data behind the technology, allows legal experts and policymakers across both governments and industry to ensure their guidance is current and relevant, and it allows other AI researchers to make further developments and decisions based on a thorough understanding of what has come before. Ethical considerations and discussion, as well as topics on any of the above are welcome in the Artificial Intelligence and Machine Learning Gateway.

The F1000Research model is particularly well-suited to facilitate the open, rapid dissemination of research works related to AI and related fields, and its applications.

Firstly, following the high-level policy decisions made by many funders already, it is essential to have a publication venue that already complies with their new requirements, and has done for many years ( Pencheva *et al*., 2018). This ensures that publications meeting these requirements are handled smoothly and expertly by F1000Research’s internal editors, who will guide authors through ensuring they comply with their funding mandates.

Secondly, trustworthy data for AI is of utmost importance to ensure accurate and efficient machine learning strategies ( Liang *et al*., 2022). While outliers or ‘untrustworthy’ data is less important in human education, due to our ability to recognize oddities or issues with datasets, AI takes all data at face value unless taught otherwise. Therefore, clean data is especially important when creating new AI software for new applications. Fostering the sharing of data that has already been analyzed and understood, and potentially used to train AI, is a cornerstone of the F1000Research model and why this Gateway has the potential to be a go-to resource for AI scholars and industry professionals alike.

Finally, most current publication venues are limited in publications to technical or applied research and excluding relevant contributions from the humanities and social sciences. The Artificial Intelligence and Machine Learning Gateway has the scope and interdisciplinary nature required to foster important sociological and other humanities research as well, providing a space where important discussions can occur about the ethics, legality and social aspects of new AI-powered technologies.

Therefore, the Artificial Intelligence and Machine Learning Gateway will provide an Open Science space for interdisciplinary publications on all aspects of AI, bridging the many different communities to create open and thoughtful discussions. It can provide a useful resource and collaborative space to guide researchers, authors and industry professionals through this new era of artificial intelligence.

## Data Availability

No data are associated with this article.
